# Responsiveness to change in health status of the EQ-5D in patients treated for depression and anxiety

**DOI:** 10.1186/s12955-023-02116-y

**Published:** 2023-04-15

**Authors:** Kenneth Sandin, Gemma Shields, Ragne G.H. Gjengedal, Kåre Osnes, Marianne T. Bjørndal, Silje E. Reme, Odin Hjemdal

**Affiliations:** 1 Division of Mental Health and Substance Abuse, Diakonhjemmet Hospital, Postboks 23 Vinderen, Oslo, 0319 Norway; 2 Department of Psychology, Norwegian University of Science and Technology, Trondheim, NO-7491 Norway; 3 Manchester Centre for Health Economics, Division of Psychology and Mental Health, School of Health Sciences, Faculty of Biology, Medicine and Health, University of Manchester, Manchester, England; 4 Department of Psychology, University of Oslo, Oslo, Norway

**Keywords:** Self-rated health, Depression, Anxiety, EQ-5D-5L, Responsiveness

## Abstract

**Background:**

The EQ-5D is a commonly used generic measure of health but evidence on its responsiveness to change in mental health is limited. This study aimed to explore the responsiveness of the five-level version of the instrument, the EQ-5D-5 L, in patients receiving treatment for depression and anxiety.

**Methods:**

Patient data (*N* = 416) were collected at baseline and at end of treatment in an observational study in a Norwegian outpatient clinic. Patients were adults of working age (18–69 years) and received protocol-based metacognitive or cognitive therapy for depression or anxiety according to diagnosis. Responsiveness in the EQ-5D was compared to change in the Beck Depression Inventory-II (BDI-II) and the Beck Anxiety Inventory (BAI). Effect sizes (Cohen’s *d*), Standardised response mean (SRM), and Pearson’s correlation were calculated. Patients were classified as “Recovered”, “Improved”, or “Unchanged” during treatment using the BDI-II and the BAI. ROC analyses determined whether the EQ-5D could correctly classify patient outcomes.

**Results:**

Effect sizes were large for the BAI, the BDI-II, the EQ-5D value and the EQ VAS, ranging from *d* = 1.07 to *d* = 1.84. SRM were also large (0.93-1.67). Pearson’s correlation showed strong agreement between change scores of the EQ-5D value and the BDI-II (*r*_*s*_ -0.54) and moderate between the EQ-5D value and the BAI (*r*_*s*_ -0.43). The EQ-5D consistently identified “Recovered” patients versus “Improved” or “Unchanged” in the ROC analyses with AUROC ranging from 0.72 to 0.84.

**Conclusion:**

The EQ-5D showed good agreement with self-reported symptom change in depression and anxiety, and correctly identified recovered patients. These findings indicate that the EQ-5D may be appropriately responsive to change in patients with depression and anxiety disorders, although replication in other clinical samples is needed.

## Abstract (summary)

The EQ-5D is a questionnaire that people fill in to report their subjective health. It is often used in clinics or hospitals to better understand how patients are affected by their illnesses, and if their health improves after treatment. For this information to be trustworthy, we need to verify how accurately the EQ-5D measures health for the particular patients we want to use it with. This is often done by comparing EQ-5D scores with scores from other questionnaires. For example, if we want to use the EQ-5D with a group of patients with depression, we compare the scores of the EQ-5D with scores from questionnaires that are commonly used to measure depression symptoms.

In this study, we compared the scores of the EQ-5D with scores from questionnaires measuring symptoms of depression and anxiety. Their performances were similar, and the EQ-5D scores could also correctly identify which patients had recovered during treatment. This implies that the EQ-5D can be a useful tool for understanding the impact of depression and anxiety and can help in decision-making regarding these patients.

## Introduction

One out of every two people will experience a mental health problem during their lifetime and mental ill health is a leading cause of global disease burden [1]. Between 2010 and 2030, mental illness is projected to cost $ 16.1 trillion worldwide, putting it on par with cardiovascular disease [2]. Depression and anxiety disorders account for 40.5% and 14.6% of the disability-adjusted life-years that are due to mental illness, making them the most costly mental health problems [3]. This substantial burden may still be underestimated [4], in part because of the wide ranging effects these disorders have on health and functioning [5].

At the recommendation of decision-making bodies such as the National institute of Health and Care excellence (NICE), generic measures are increasingly used to capture health status [6]. Mental disorders like depression and anxiety have broad, negative impact on quality of life and wellbeing that may not be adequately reflected by condition-specific measures [5, 7]. Generic measures may thus be a valuable supplement to measures of primary symptoms as they capture a broader measure of health. These instruments can also be used to compare burden of disease and impact of interventions between different patient groups, such as in cost-benefit analyses, making them useful tools for decision-makers, researchers, and clinicians [8]. To adequately fill this role, it must be demonstrated that the generic measure in question can accurately capture health status in the relevant patient population.

One of the most commonly used generic measures of health-related quality of life is the EQ-5D [8]. The EQ-5D records health status across five dimensions: Mobility, Self-care, Usual activities, Pain / discomfort, and Anxiety / depression [9]. The previous version of the EQ-5D, the EQ-5D-3L, used three levels of severity and showed good psychometric properties in depression, but mixed results in anxiety disorders [10]. A recent review evaluated the properties of the newer five-level version of the EQ-5D, the EQ-5D-5L, across multiple patient groups [11]. These and other studies of patients with mental health problems have shown moderate to good correlation between condition-specific measures and the EQ-5D-5L in cross-sectional designs [11–15].

These studies did not include data on responsiveness [11]. Responsiveness is often defined as an instruments ability to detect clinically significant change over time [10, 16]. Two criteria have been suggested for defining what constitutes “clinically significant change”: That the magnitude of change be statistically reliable, and that patients end up in a clinical range that renders them indistinguishable from the normal population, i.e. they have recovered [17]. Responsiveness according to these criteria is not a fixed parameter, but will likely vary according to populations and context [18]. This makes it necessary to investigate responsiveness across multiple patient groups. One study did find reasonable validity and moderate responsiveness in anxiety on the EQ-5D-3L [19]. But only a few studies have examined this aspect of the five-level EQ-5D-5L in depression and anxiety [11].

One study found that using only the Anxiety/depression dimension of the EQ-5D-5L did not adequately capture responsiveness in anxiety and depression for patients treated in a general internal medicine ward [20]. Another study found that the EQ-5D-5L could adequately screen for depression and anxiety by distinguishing between severity levels in patients with type 2 diabetes. This was true of both the Anxiety / depression dimension and the EQ-5D value [21]. However, this was a cross-sectional design, and the ability of the EQ-5D-5 L to detect change in severity over time in patients with depression and anxiety is not established, and was specifically targeted by a review of the literature as a future research priority [11]. Investigating this aspect of the EQ-5D-5 L is imperative in establishing whether it is a valid tool for capturing the health status of these patients.

To our knowledge, ours is the first study to examine the responsiveness of the EQ-5D-5L in patients treated for depression and anxiety as their primary diagnoses. In line with recommendations and methodology used in previous studies, we explored responsiveness of the EQ-5D-5L by comparing change from start to end of intervention with change in condition-specific measures [17, 20, 21] The aim of the study was thus to test the following hypotheses: (1) that the EQ-5D-5L shows similar range in effect size and an at least moderate correlation with change scores in condition-specific measures, and (2) that the EQ-5D-5L can identify patients classified as “Recovered” by condition-specific measures at end of treatment.

## Methods

### Study context

Data were collected in a naturalistic observational study that ran from May 2017 – March 2020 at the Department of Mental Health and Substance Abuse, Diakonhjemmet Hospital in Oslo, Norway. The clinic is part of the national health service, and the study is part of the project “The Norwegian studies of psychological treatments and work (NOR-WORK)”. Patients are referred by their general practitioners for treatment of depression and anxiety. Patients at the clinic are generally of working age, and previous research has shown that on average, half the patients are on sick leave due to depression or anxiety at baseline [22]. They are then screened by a clinical psychologist using anamnestic information, the Beck Depression Inventory-II (BDI-II), the Beck Anxiety Inventory (BAI), and the MINI-International Neuropsychiatric Interview [23–25]. Patients are diagnosed during the screening in accordance with the International Classification of Diseases 10 (ICD-10) [26]. Inclusion criteria for the present study were that the patient was an adult of working age (18–70 years) with clinically significant levels of depression and anxiety operationalised as follows: Patients with a primary depression diagnosis had to have a minimum score of 14 on the BDI-II, and patients with a primary anxiety diagnosis had to have a minimum score of 16 on the Beck Anxiety Inventory BAI. In addition to primary depression or anxiety diagnoses, patients with adjustment disorder and mixed anxiety and depression were included in the study. Adjustment disorder is sometimes referred to as “situational depression”, underlining its close relationship with depressive disorders [26]. Similarly, patients with a mixed anxiety and depressive disorder were included as the diagnosis is comprised of symptoms of anxiety and depression.

Exclusion criteria were severe mental illness such as bipolar disorder, high risk of suicide, engaging in active substance abuse, or suffering from cluster A or B personality disorder. Patients scoring below clinical thresholds for depression and anxiety on the BDI and BAI at baseline were excluded from the study. All patients who signed a written consent form and completed treatment, including filling in questionnaires at baseline and at end of treatment, were included (*N* = 416). The current study thus focused on patients who completed treatment.

Patients received either Metacognitive therapy (MCT) or Cognitive behavioural therapy (CBT) according to diagnose-specific manuals [27, 28], and average duration of treatment was 10.11 sessions (SD 3.93). Previous research has shown that half the patients are on sick leave when referred, and treatment thus also includes interventions aimed at helping patients return to work [29].

### Instruments

Clinical and sociodemographic data were collected at baseline and end of treatment from patient journals and from self-report questionnaires.

*The EQ-5D-5L*: The EQ-5D-5L questionnaire firstly asks respondents to rate their current health on five dimensions: Mobility, Self-Care, Usual activities, Pain / discomfort, and Anxiety / depression on a severity scale from 1 (“No problems”) to 5 (“Severe problems”). The combined severity ratings give an EQ-5D profile, e.g. “11111” in the case of “No problem” on all five dimensions. This health profile can be converted to the EQ-5D value using preference-based weights. A value of 0.00 indicates death and 1.00 indicates perfect health. The EQ-5D value can be used to calculate quality-adjusted life-years (QALYs), i.e. a score of 1.00 for one year equals one QALY. The preference-based weights used to convert responses to EQ-5D values are often referred to as “value sets”. A study is underway, but there is currently no Norwegian value set [30]. This study used the crosswalk system recommended by NICE for converting EQ-5D profiles to EQ-5D values [31, 32]. For the EQ-5D value, healthy people generally report scores close to 1.0. In a recent survey of the Norwegian general population, the mean EQ-5D value in a postal survey was 0.848 [33].

The second part of the EQ-5D-5L asks patients to rate their health on a 20 cm visual analogue scale (VAS) where the bottom (“0”) indicates worst imaginable health, and the top (“100”) indicates best imaginable health. Although it is related to the EQ-5D profile and the value scores, it does not measure the same construct. For instance, the EQ VAS score has been shown to decline with age even for people whose EQ-5D profile show no problems (“11111”) [8].

*The Beck Depression Inventory-II (BDI-II)* is a 21-item questionnaire measuring severity of symptoms over the last two weeks on a scale from 0 to 3, giving a total sum score of 0–63. Examples include feeling sad and change in appetite or sleep. Suggested scoring indicates that 0–13 reflects minimal symptoms, 14–19 mild, 20–28, moderate, and 29–63 severe symptoms [24]. The BDI-II has been found to be psychometrically sound in depression[31], Chronbach’s *α* in the current study was 0.86.

*The Beck Anxiety Inventory (BAI)* is a self-report measure of anxiety severity over the last week. As with the BDI-II, anxiety symptoms (e.g. “Heart pounding or racing” or feeling “nervous”) are scored on a severity range from 0 to 3, giving a total sum score of 0–63. Suggested scoring indicates that 0–15 reflects mild symptoms, 16–25 moderate, and 26–63 severe symptoms. The BAI has demonstrated good psychometric properties [34], Chronbach’s *α* in the current study was 0.90.

### Statistical analyses

Descriptive statistics on age, gender, education level and diagnosis were compiled at baseline. Distribution of scores on the EQ-5D dimensions were calculated in percentages at baseline and at end of treatment and analysed using a non-parametric test of trends developed by Cuzick. The test is similar to the Wilcoxon rank-sum test [35]. Mean scores and standard deviations at baseline and end of treatment, including change (∆) during treatment, were calculated for the BAI, the BDI-II, the EQ-5D values, and the EQ VAS. Effect sizes (ES) were calculated from baseline to end of treatment using Cohen’s *d.* Values < 0.5 are considered small, ≥ 0.5 < 0.8 moderate, and ≥ 0.8 large [36]. We also calculated the standardised response mean (SRM), defined as the mean change in score from baseline to end of treatment divided by the standard deviation of change in scores [37]. For the SRM it is suggested that magnitude of change is dependent on correlation between scores at baseline and end of treatment. For example, SRM > 0.8 can be interpreted as large if this correlation < 0.5, moderate if correlation > 0.5 [38]. Agreement between the change scores on the four measures were also analysed with Pearson’s correlation. Pearson’s correlation < 0.40 are considered weak, 0.40–0.49 moderate, and > 0.50 are considered strong [39].

Using the BAI and the BDI-II, the patients were then classified according to treatment response. With a minimum score of 14 on the BDI-II for depression patients and 16 on the BAI for anxiety patients at baseline, based on scoring norms for the BDI-II and BAI, patients were classified thus: “Deteriorated” if their scores increased by 9 points or more from baseline to end of treatment, “Unchanged” if the change was less than 9 points in either direction, and “Improved” if the scores decreased by 9 points or more but score at the end of treatment was still above the clinical threshold, . Finally, patients were classified as “Recovered” if their score decreased by 9 points or more and their final score was below clinical threshold (i.e. 14 for the BDI-II and 16 for the BAI) [18, 40, 41].

We ran ROC curve analyses to determine how well the EQ-5D value scores could correctly classify patients according to the clinical criteria of the BDI-II and the BAI: Recovered versus Improved, Recovered versus Unchanged, and Improved versus Unchanged. Analyses of BDI were run to calculate the area under the curve (AUROC) using the entire sample for patients that had a BDI score of at least 14 at baseline, and for all patients who had a BAI score of at least 16, regardless of primary diagnoses. Then, using primary diagnosis as recorded from the medical journals, we then calculated the AUROC for BDI-II for only the patients with depression as primary diagnosis and BDI-II baseline scores of at least 14. Lastly, we calculated the AUROC for BAI for the patients with anxiety as their primary diagnosis and a BAI baseline score of at least 16. The EQ-5D value at end of treatment was used as classifier, when computing the AUROC. AUROC was interpreted as < 0.50 useless test, 0.51–0.69 poor test, 0.7–0.79 fair test, 0.8–0.89 good test, 0.9–0.99 excellent test, 1.0 perfect test [40]. We calculated the sample size needed for the groups included in the ROC analyses. We set the Alpha level to 0.05 and the Beta level to 0.20, area under curve was set to 0.7 and value of null hypothesis was set to 0.5. The ratio of positive to negative cases was set according to the characteristics of the sample. We also computed cut-off values for recovery using Youden’s index (J), which displays which values have the highest combined sensitivity and specificity [42].

Generally accepted methods for handling missing data are applicable to the EQ-5D-5L [8]. Missing data on individual items in the current study were replaced by weighted means, a method developed for treating missing data in depression cohorts [43]. All analyses were carried out using STATA 16 [44].

### Ethical considerations

All patients included in the study gave written, informed consent to participate. The study is classified as health service research under Norwegian regulation. The Norwegian Data Protection Agency has in such cases designated that treatment providers (i.e. hospitals) are responsible for proper data management. Data collection and security in the present study was managed by Diakonhjemmet Hospital, and approval of data handling was granted by Oslo University Hospital, approval number 2015/15606. The study was carried out in accordance with the principles of the Helsinki declaration.

## Results

Characteristics of included patients (*N* = 416) at baseline are shown in Table 1. Average age of patients was 37.7 years, the youngest was 18 and the oldest 65 years at start of treatment. Females made up 71.9% of the patient sample, which is in line with the gender disparity seen in prevalence studies of depression and anxiety [45]. More than 80% of the sample had some form of higher education. The study only recorded primary diagnosis from the patient’s medical journal, but comorbidity was not recorded. The majority of patients had either a primary depression or anxiety diagnosis, the remaining patients were diagnosed with either mixed anxiety / depression, or adjustment disorder. The most prevalent single diagnoses were F32 Major depressive disorder, single episode (*n* = 114, 26.8%), F 33 Major depressive disorder, recurrent (*n* = 97, 22.8%), and F 41.1 Generalised anxiety disorder (*n* = 86, 20.2%). Missing data in the study was typically low, > 5% on individual items for all measures.

**Table 1:** Demographic characteristics and diagnoses of patients at baseline (*N* = 416)

				Mean		SD			*n*		*%*
Age, years				37.66	10.65						
Gender											
	Female Male							299		71.88	
								117		28.13	
Education											
	Primary / Secondary Higher education ≤ 4 yrs Higher education > 4 yrs							70		17.16	
								151		37.01	
								187		45.83	
Primary diagnosis											
	Depression disorder Anxiety disorder Mixed anxiety / depression Adjustment disorder							216		51.92	
								161		38.70	
								24		6.77	
								15		3.61	

### Change in depression, anxiety and the EQ-5D-5 L during treatment

Of the 216 patients with depression diagnoses, 146 (67.59%) were “Recovered” at end of treatment, 31 (14.35%) were “Improved”, and 39 were (18.05%) were “Unchanged”. Of the 161 patients with anxiety disorder diagnoses, 109 (67.70%) were “Recovered” at end of treatment, 14 (8.69%) were “Improved”, and 38 were (23.60%) were “Unchanged”. Overall, two patients in the sample were “Deteriorated” on the BAI at end of treatment, both were diagnosed with adjustment disorder. Four patients were “Deteriorated” on the BDI-II, three of which were diagnosed with adjustment disorder, and one with anxiety disorder. No patients with anxiety diagnoses were “Deteriorated” on the BAI at end of treatment, and no patients with depression diagnoses were “Deteriorated” on the BDI-II at end of treatment.

Table 2 shows the distribution of scores on the EQ-5D dimensions at baseline, and after end of treatment. All dimensions had at least some patients reporting problems at baseline. Cuzick’s non-parametric test of trends showed that all dimensions saw significant improvement from baseline to end of treatment [33]. The symptom scores reported on the BDI-II and the BAI at baseline in Table 3 indicate moderate levels of depression and anxiety. Patients saw a marked improvement in symptoms over the observation period. Cohen’s *d* was > 0.8 on all measures from baseline to end of treatment. Similarly, all SRM showed values > 0.8 on all instruments. Correlation between baseline scores and scores at end of treatment were < 0.5 on the BDI-II (*r*_*s*_ = 0.39), EQ-5D value (*r*_*s*_ = 0.34), and the EQ-VAS (*r*_*s*_ = 0.31), but > 0.5 on the BAI (*r*_*s*_ = 0.51). This indicates that the SRM was large for the BDI-II, EQ-5D value, and the EQ VAS, whilst moderate for the BAI.

**Table 2:** Distribution of EQ-5D dimensions as reported by patients (*N* = 416)

				Baseline			End of treatment			*p* value
				*n*	*%*		*n*	*%*		
Mobility										
	No problems Slight problems Moderate problems Severe problems Unable to walk about			323	78.59		364	88.35		< 0.001
				62	15.09		39	9.47		
				20	4.87		9	2.18		
				6	1.46		.	.		
				.	.		.	.		
Self-care										
	No problems Slight problems Moderate problems Severe problems Unable to wash or dress			350	85.37		398	96.60		< 0.001
				52	12.68		11	2.67		
				6	1.46		2	0.49		
				2	0.49		1	0.49		
				.	.		.	.		
Usual activities										
	No problems Slight problems Moderate problems Severe problems Unable to do usual activities			83	20.19		258	62.93		< 0.001
				161	39.17		111	27.07		
				113	27.49		35	8.54		
				50	12.17		6	1.46		
				4	0.97		.	.		
Pain / discomfort										
	No problems Slight problems Moderate problems Severe problems Extreme pain or discomfort			93	22.63		206	50.00		< 0.001
				177	43.07		151	36.65		
				113	27.49		45	10.92		
				24	5.84		7	1.70		
				4	0.97		3	0.73		
Anxiety / depression										
	No problems Slight problems Moderate problems Severe problems Extremely anxious or depressed			13	3.17		160	38.83		< 0.001
				106	25.85		187	44.39		
				179	43.66		53	12.86		
				105	25.61		11	2.67		
				7	1.71		1	0.24		

Note. Proportion of patients reporting the various levels of severity on the EQ-5D dimensions at baseline, and at end of treatment. The *P*-values denote significance of a non-parametric test of trends across ordered groups, developed by Cuzick, similar to the Wilcoxon rank-sum test [35]

**Table 3:** Instrument scores at baseline and end of treatment with ES and SRM (*N* = 416)

		Baseline				End of treatment				*d*	SRM
		*Mean*	*SD*			*Mean*		*SD*			
Anxiety (BAI)		18.48		10.27		6.49	6.47			1.39	1.35
Depression (BDI-II)		26.42		8.77		10.13	8.84			1.84	1.67
EQ-5D value		0.630		0.189		0.816	0.153			1.07	0.93
EQ VAS		54.58		17.13		74.71	14.80			1.25	1.06

Note. Abbreviations: BAI, the Beck Anxiety Inventory; BDI-II, the Beck Depression Inventory-II; ES, Effect Size (reported in Cohen’s *d*); SRM, Standardised response mean

### Correlation of change scores

Pearson’s rank order correlations are shown in Table 4. Note that the BAI and the BDI-II indicate worse health status with higher scores, whereas the reverse is true for the EQ-5D value and the EQ VAS. The EQ-5D value showed strong correlations with both the BDI-II, the EQ VAS, and moderate correlations with the BAI. The EQ VAS showed strong correlation with the BDI-II, but weak correlation with the BAI.

**Table 4:** Pearson’s correlation of change scores (*N* = 416)

	BAI ∆	BDI-II ∆	EQ-5D value ∆
BAI ∆			
BDI-II ∆	0.49		
EQ-5D value ∆	-0.43	-0.54	
EQ VAS ∆	-0.34	-0.58	0.55

Note. Change scores calculated by subtracting the score at end of treatment from the score at baseline. Abbreviations: BAI, the Beck Anxiety Inventory; BDI-II, the Beck Depression Inventory-II. Pearson’s correlation < 0.40 are considered weak, 0.40–0.49 moderate, and > 0.50 are considered strong [37]

### ROC curve analysis

For the total sample, the ROC curve analysis showed that the EQ-5D value consistently distinguished between “Recovered” and “Improved” or “Unchanged” patients according the BDI-II or BAI, AUROC ranging from 0.72 to 0.84 (Table 5). The AUC did not adequately distinguish between “Improved” and “Unchanged” on either measure, AUROC ranged from 0.49 to 0.61.

**Table 5:** Area under the receiver operating characteristic curve (AUROC) using non-parametric ROC analyses (*N* = 416)

							BDI-II					BAI			
					n	C-statistic		SE	95% CI		n		C-statistic	SE	95% CI
Overall															
	Recovered vs. Unchanged Recovered vs. Improved Improved vs. Unchanged				329	0.81		0.032	(0.75–0.88)		215		0.82	0.039	(0.75–0.90)
					314	0.78		0.035	(0.71–0.85)		210		0.72	0.061	(0.60–0.84)
					121	0.52		0.052	(0.48–0.68)		49		0.64	0.079	(0.49–0.80)
Depression															
	Recovered vs. Unchanged Recovered vs. Improved Improved vs. Unchanged				184	0.80		0.045	(0.71–0.88)						
					176	0.75		0.047	(0.65–0.84)						
					68	0.61		0.070	(0.47–0.75)						
Anxiety															
	Recovered vs. Unchanged Recovered vs. Improved Improved vs. Unchanged										147		0.83		(0.71–0.95)
											123		0.84		(0.70–0.97)
											52		0.53		(0.29–0.77)

Note. Area under the curve (AUROC) reported for receiver operating analyses. The BAI and the BDI-II were used as classifiers of whether patients were “Recovered”, “Improved”, or “Unchanged” at end of treatment. The “Overall” analyses were run using the BAI and the BDI-II for the whole sample regardless of primary diagnosis, the “Depression” analyses were run using the BDI-II for only the patients with depression as primary diagnosis, and the “Anxiety” using the BAI for only the patients with anxiety as primary diagnosis. Abbreviations: BAI, the Beck Anxiety Inventory; BDI-II, the Beck Depression Inventory-II.

### < Table 5 APPROXIMATELY HERE>

The same pattern repeated when patients scores were analysed according to diagnoses. For patients with depression, the AUC was excellent when distinguishing between “Recovered” and “Unchanged” (0.81) and acceptable distinguishing “Recovered” from “Improved” (0.78), but ineffective separating “Improved” and “Unchanged” (0.52). For patients with anxiety, the AUC showed excellent classification for “Recovered” versus “Unchanged” (0.83). Our analyses of “Recovered” versus “Improved” and “Improved” versus “Unchanged” did not have appropriate statistical power and can thus not be regarded as significant findings. Youden’s index indicated that an EQ-5D value of 0.768 had the highest combined sensitivity and specificity when identifying recovered patients in the total sample. The value was the same for both depression and anxiety (Table 6).

**Table 6:** The central range of operating characteristics of the EQ-5D value post-treatment for identifying recovered versus non-recovered patients (*N* = 416)

	EQ-5D cut-off				Sensitivity		Specificity		*J*		Correctly classified
Depression											
		0.740			84.72%		62.69%		0.47		77.73
		0.750			81.25%		62.69%		0.44		75.36
		**0.768**			**80.56%**		**64.18%**		**0.45**		**75.36**
		0.819			74.31%		67.16%		0.41		72.04
		0.827			74.31%		68.66%		0.43		72.51
Anxiety											
		0.740			84.75%		80.00%		0.65		83.76
		0.750			83.70%		80.00%		0.64		82.91
		**0.768**			**83.70%**		**84.00%**		**0.68**		**83.76**
		0.819			63.04%		84.00%		0.47		67.52
		0.827			60.87%		84.00%		0.45		65.81

Note. *J* = Youden’s index. Depression and Anxiety recovery measured and classified using the BDI-II and the BAI, respectively

## Discussion

Our aim was to explore the responsiveness of the EQ-5D-5L in patients receiving treatment for depression and anxiety. This was done by comparing change in the EQ-5D-5L to change in the disorder-specific measures BDI-II and BAI. We hypothesised that the EQ-5D-5L should show magnitude of change as the BDI-II and BAI during treatment. The ES was large (*d* > 0.8) for all measures, ranging from Cohen’s *d* 1.07–1.84. For the SRM, which accounts for variability in treatment response by dividing change scores by the standard deviation of change scores, the BDI-II, the EQ-5D value and the EQ VAS all showed large magnitude of change. The BAI showed moderate magnitude of change on the SRM when accounting for its higher correlation between baseline and end of treatment scores. Furthermore, the EQ-5D-5L change scores showed strong correlation with the BDI-II, and moderate correlation with the BAI. The hypothesis that the EQ-5D-5 L should show similar magnitude of change as the condition-specific measures thus seems confirmed.

We then examined if the EQ-5D value could correctly classify patients deemed as “Recovered” according to the condition-specific measures. Results from the ROC analyses indicate that this was the case: AUROC were from fair to good when distinguishing “Recovered” patients from “Improved” or “Unchanged”. This was true for the total sample (AUROC 0.72–0.82), for patients with depression (AUROC 0.75 and 0.80), and for patients with anxiety when distinguishing “Recovered” patients from “Unchanged” patients (AUROC 0.83). In a similarly consistent pattern, the EQ-5D-5L showed poor ability to distinguish between “Improved” and “Unchanged” patients for the total sample, for depression, and for anxiety, (AUROC 0.52–0.64). The ability of the EQ-5D-5L to consistently identify recovered patients indicates that our second hypothesis was confirmed. We also calculated Youden’s index, as this may be informative for clinicians and serve as a reference for future research. For recovery from both depression and anxiety in the total sample, cut-off point as defined by highest combined sensitivity and specificity was an EQ-5D value ≥ 0.768 at end of treatment.

Data on the responsiveness of the five-level version of the EQ-5D-5L in mental health is limited, though cross-sectional measures have indicated moderate to good correlation with condition-specific measures [11]. Comparing to the three-level version, one study found moderate responsiveness to anxiety disorders. Similar to the present study, patients were classified as having either “more”, “constant”, or “less anxiety” according to the BAI. T-tests showed significant differences in change scores for the EQ-5D value and the EQ VAS. However, that study found that the SRM were moderate to small, and ES were large for the EQ-5D value only when patients were deteriorated [19].

Reviews of the literature on the three-level version have indicated reasonable responsiveness in depression and anxiety [10], suggesting that the five-level version may have similar properties. One recent study compared the responsiveness of the three-level and five-level versions of Anxiety / depression dimension for mental health patients. Although the five-level version was found to be more responsive, both showed limited ability to capture changes in mental health [20]. The Anxiety / depression dimension did show significant change from baseline to end of treatment in the present study. Future research may determine how useful it is as a measure on its own.

A previous cross-sectional study did find that the EQ-5D value could screen for depression and anxiety in patients with type 2 diabetes [21]. In the present study, the EQ-5D value showed similar performance in a longitudinal design in patients with depression and anxiety as primary diagnoses. That the EQ-5D value may perform better than the Anxiety / depression dimension alone is perhaps reasonable, as it may better capture the wide-ranging impact of depression and anxiety on health and quality of life [4, 5].

The EQ-5D-5L is increasingly used when evaluating health status in surveys and clinical trials [8], and decision-making bodies recommend its use in evaluating health technologies [6, 46]. Demonstrating its validity in diverse patient groups is therefore essential for sound decision-making when allocating healthcare resources. In this study, the EQ-5D-5L showed good responsiveness to change for patients with depression and anxiety. This suggests that the EQ-5D-5L can be a valid and useful tool for evaluating impact of disease and benefit of treatment for these patients, for instance through estimating QALYs. It also suggests that the EQ-5D-5L can useful when evaluating interventions for patients with depression and anxiety.

### Strengths and limitations

The main strength of the study is adding to a limited evidence-base concerning the responsiveness of the five-level version of the EQ-5D in patients with depression and anxiety. The study included a fairly large clinical sample who were assessed and diagnosed by clinical psychologists before entering treatment. We can thus be reasonably certain of the clinical characteristics of the sample. The study took part in a national health service clinic, suggesting that these patients are somewhat representative of clinical populations with depression and anxiety in Norway. The patients saw substantial treatment gains as reflected by the large ES and SRM, which gave an opportunity for evaluating the ability of the EQ-5D-5L to identify recovered patients.

Several limitations to the study have to be considered. The study only included patients who completed treatment, and treatment gains were large. The study could therefore not evaluate the ability of the EQ-5D-5L to detect smaller changes, that still may be of importance to patients. A related limitation is that the large rate of recovered patients in the study meant that “Unchanged” patients formed a small subgroup. The findings concerning the unchanged patients should be treated with caution. We also lack adequate data to determine if the EQ-5D-5L would be equally responsive to deterioration as improvement during treatment. The study also lacked data on comorbidity.

The current study uses the UK value set for converting to EQ-5D value scores, as there is currently no Norwegian value set available. Choice of value sets has shown to influence the estimation of QALYs, which suggests that it would be useful to replicate the present findings when a Norwegian value set is available [14].

As new measures of health status become available, such as the Recovering Quality of Life (ReQoL), it will be important to compare and contrast these against the EQ-5D-5L to judge which instrument is best suited for patients with depression and anxiety [47]. There is evidence that a wide range of outcomes that are important to patients with mental health problems are not adequately captured by commonly used instruments [5, 7]. Further research is needed to assess whether the EQ-5D-5L could reflect key changes in a wider range of outcomes [5], or if other instruments or bolt-on dimensions may be better for capturing psycho-social factors of importance to patients [48].

## Conclusion

The findings in this study suggest that the EQ-5D-5L may be responsive to change in health status for patients receiving treatment for depression and anxiety. The EQ-5D-5L showed similar magnitude of change as the condition-specific measures and was also able to consistently identify patients who had recovered from depression and anxiety. Responsiveness of the EQ-5D-5L is likely sensitive to context, and these findings should be replicated in other samples. Still, these findings suggest that the EQ-5D-5L may be a useful tool for evaluating outcomes of treatment for patients with depression and anxiety.

## Data Availability

material. The patient data used for this study is not readily available as the patients have not given consent for use or distribution beyond the research at Diakonhjemmet Hospital. Inquiries can be directed to kenneth.sandin@diakonsyk.no.

## Code Availability

Any inquiry can be directed to kenneth.sandin@diakonsyk.no.
